# Genotyping of potato samples from the GenAgro ICG SB RAS collection using DNA markers of genes conferring resistance to phytopathogens

**DOI:** 10.18699/VJ21.077

**Published:** 2021-10

**Authors:** I.V. Totsky, I.V. Rozanova, A.D. Safonova, A.S. Batov, Yu.A. Gureeva, E.K. Khlestkina, A.V. Kochetov

**Affiliations:** Institute of Cytology and Genetics of the Siberian Branch of the Russian Academy of Sciences, Novosibirsk, Russia; Siberian Research Institute of Plant Production and Breeding – Branch of the Institute of Cytology and Genetics of the Siberian Branch of the Russian Academy of Sciences, Novosibirsk, Russia; Institute of Cytology and Genetics of the Siberian Branch of the Russian Academy of Sciences, Novosibirsk, Russia; Federal Research Center the N.I. Vavilov All-Russian Institute of Plant Genetic Resources (VIR), St. Petersburg, Russia; Siberian Research Institute of Plant Production and Breeding – Branch of the Institute of Cytology and Genetics of the Siberian Branch of the Russian Academy of Sciences, Novosibirsk, Russia; Siberian Research Institute of Plant Production and Breeding – Branch of the Institute of Cytology and Genetics of the Siberian Branch of the Russian Academy of Sciences, Novosibirsk, Russia; Siberian Research Institute of Plant Production and Breeding – Branch of the Institute of Cytology and Genetics of the Siberian Branch of the Russian Academy of Sciences, Novosibirsk, Russia; Institute of Cytology and Genetics of the Siberian Branch of the Russian Academy of Sciences, Novosibirsk, Russia; Federal Research Center the N.I. Vavilov All-Russian Institute of Plant Genetic Resources (VIR), St. Petersburg, Russia; Institute of Cytology and Genetics of the Siberian Branch of the Russian Academy of Sciences, Novosibirsk, Russia

**Keywords:** golden potato cyst nematode; ; ; ; ; ;, wart, potato, DNA markers 57R, NL25, CP113, Gro1-4, золотистая картофельная нематода, рак картофеля, картофель, ДНК-маркеры 57R, NL25, CP113, Gro1-4

## Abstract

Wart (a disease caused by Synchytrium endobioticum) and golden cyst potato nematode (Globodera rostochiensis), which parasitize the roots of the host plant, cause signif icant damage to potato crop. Both of these disease factors are quarantined in the Russian Federation, and each registered variety is tested for resistance to their most common races and pathotypes. The main method of opposing such diseases is by the development of resistant varieties. An important step in this process is the selection of resistant genotypes from the population and the estimation of the resistance of hybrids obtained by crosses during the breeding process. Conducting a permanent phenotypic evaluation is associated with diff iculties, for example, it is not always possible to work with pathogens, and phenotypic evaluation is very costly and time consuming. However, the use of DNA markers linked to resistance genes can signif icantly speed up and reduce the cost of the breeding process. The aim of the study was to screen the GenAgro potato collection of ICG SB RAS using known diagnostic PCR markers linked to golden potato cyst nematode and wart resistance. Genotyping was carried out on 73 potato samples using three DNA markers 57R, CP113, Gro1-4 associated with nematode resistance and one marker, NL25, associated with wart resistance. The genotyping data were compared with the data on the resistance of the collection samples. Only the 57R marker had a high level of correlation (Spearman R = 0.722008, p = 0.000000, p < 0.05) between resistance and the presence of a diagnostic fragment. The diagnostic eff iciency of the 57R marker was 86.11 %. This marker can be successfully used for screening a collection, searching for resistant genotypes and marker-assisted selection. The other markers showed a low correlation between the presence of the DNA marker and resistance. The diagnostic eff iciency of the CP113 marker was only 44.44 %. Spearman’s correlation coeff icient (Spearman R = –0.109218, p = 0.361104, p < 0.05) did not show signif icant correlation between resistance and the DNA marker. The diagnostic eff iciency of the NL25 marker was 61.11 %. No signif icant correlation was found between the NL25 marker and resistance (Spearman R = –0.017946, p = 0.881061, p < 0.05). The use of these markers for the search for resistant samples is not advisable.

## Abstract (ru)

Значительный ущерб урожаю картофеля наносят рак картофеля (болезнь, вызываемая патогенным грибом Synchytrium endobioticum) и золотистая картофельная нематода (Globodera rostochiensis), паразитирую- щая на корнях растения-хозяина. Оба этих фактора являются объектами внешнего и внутреннего карантина в Российской Федерации, и каждый сорт, регистрируемый в РФ, проходит проверку на устойчивость к наиболее распространенным их расам и патотипам. Основной метод борьбы с подобными заболеваниями – выведение устойчивых сортов. Важным этапом в этом процессе является отбор устойчивых генотипов из популяции и оцен- ка устойчивости гибридов, полученных при скрещиваниях во время селекционного процесса. Проведение по- стоянной фенотипической оценки связано с рядом трудностей, а именно: не всегда есть возможность работать с патогенами, сама фенотипическая оценка очень затратная и трудоемкая. Однако применение ДНК-маркеров, сцепленных с генами устойчивости, может значительно ускорить и удешевить процесс. Целью исследования было проведение скрининга коллекции картофеля «ГенАгро» (ИЦиГ СО РАН) с использованием ПЦР-маркеров, разработанных для диагностики устойчивости к золотистой картофельной нематоде и раку картофеля. Семьде- сят три образца из коллекции «ГенАгро» ИЦиГ СО РАН были генотипированы ДНК-маркерами 57R, CP113, Gro1-4, сцепленными с устойчивостью к нематоде, и маркером NL25 для устойчивости к раку. Результаты генотипиро- вания сопоставлены с уровнем восприимчивости образцов к болезням. Высокий уровень корреляции (коэффи- циент корреляции Спирмена Spearman R = 0.722008, p = 0.000000, p < 0.05) между устойчивостью и наличием диагностического фрагмента был показан только для маркера 57R. Диагностическая эффективность маркера 57R составила 86.11 %. Данный маркер можно успешно использовать для поиска устойчивых генотипов и про- ведения маркер-ориентированной селекции. Для остальных маркеров достоверных корреляций не выявлено. Диагностическая эффективность применения маркера CP113 равнялась всего 44.44 %, а коэффициент корреляции Спирмена (Spearman R = –0.109218, p = 0.361104, p < 0.05) показывал отсутствие значимой корреляция меж- ду устойчивостью и ДНК-маркером. Диагностическая эффективность маркера NL25 составила 61.11 %. Значи- мой корреляции между маркером NL25 и устойчивостью не обнаружено (Spearman R = –0.017946, p = 0.881061, p < 0.05). Использование этих маркеров для поиска устойчивых образцов нецелесообразно.

## Introduction

Potato is one of the most important crops in the world and is the world’s fifth largest staple food crop by volume (FAO Statistical Pocketbook, 2019). One of the possible reasons for a decrease in yield is the damage of potatoes by various factors. Especially dangerous for potatoes are golden potato cyst nematode (Globodera rostochiensis) and potato wart (pathogen – Synchytrium endobioticum). They are quarantined in the Russian Federation. Data on resistance to G. rostochiensis and S. endobioticum are required when registering a potato variety in the State Register of Selection Achievements Authorized for Use (State Register… 2019; https://gossortrf.ru/).

Potato cyst nematode (PCN) can cause significant damage to the potato yield, which can reach 80–90 % (Khiutti et al., 2017; Klimenko et al., 2017). Today, 5 pathotypes of this pest are known in the world: Ro1, Ro2, Ro3, Ro4, Ro5 (Kort et al., 1977; Khiutti et al., 2017), while in Russia only the Ro1 pathotype of PCN has been detected at the moment (Limantseva et al., 2014).

Potato wart affects from 35 (Koretsky, 1970) to 100 % (Hampson, 1993) of the yield. There are 43 wart pathogens in Europe today (Baayen et al., 2006). Only a few varieties affected by this disease are registered in the State Register of Selection Achievements (State Register… 2019; https:// gossortrf.ru/).

One of the main methods of dealing with these pests is the development of resistant potato varieties. Accordingly, it is important to detect genes responsible for resistance to PCN, study their heritability, develop DNA markers linked to these genes, and use genes in breeding in marker-assisted selection schemes.

The potato has 7 loci of resistance to PCN on chromosomes III (Gro1.4-QTL (Kreike et al., 1996)), V (Grp1-QTL (Rouppe van der Voort et al., 1998), H1 (Gebhardt et al., 1993), GroV1 (Pineda et al., 1993)), VII (Gro1 (Barone et al., 1990; Leister et al., 1996)), X (Gro1.2-QTL (Kreike et al., 1993)), XI (Gro1.3-QTL (Kreike et al., 1993)). Four loci (Gro1.4, Grp1, Gro1.2, and Gro1.3) provide partial resistance, while three others (H1, GroV1, and Gro1) give high resistance to one or more pathotypes (Gebhardt, Valkonen, 2001; Bakker et al., 2004; Ramakrishnan et al., 2015). DNA markers have made it possible to identify complex loci containing several R-genes, including a locus containing two genes (H1, GroV1) for PCN resistance, which was identified on chromosome V in two different potato species (Gebhardt, Valkonen, 2001).

The H1 resistance gene is introgressed into breeding varieties from Solanum tuberosum ssp. andigenum and S. vernei (Toxopeus, Huijsman, 1953). This gene is dominant and determines resistance to pathotypes Ro1 and Ro4 of G. rostochiensis (Jones et al., 1981; Gebhardt, Valkonen, 2001; Bakker et al., 2004); according to other data, it determines resistance to pathotypes Ro5 and Ro6 (Pajerowska-Mukhtar et al., 2009; Milczarek et al., 2011; Lopez-Pardo et al., 2013; Ramakrishnan et al., 2015). This gene is located at the distal part of the long arm of the V chromosome (Gebhardt et al., 1993; Pineda et al., 1993) and encodes the CC-NBS-LRR protein (coiled coil/nucleotide-binding/leucine-rich repeat). The H1 gene is the only nematode resistance gene for which Flora’s geneto- gene interaction concept has been validated by classical genetic analysis (Flor, 1971; Janssen et al., 1991; Gebhardt, Valkonen, 2001). The H1 resistance gene corresponded to the Avr gene of golden potato cyst nematode G. rostochiensis.

The GroV1 gene originates from the wild potato species S. vernei, is linked to the H1 locus (Jacobs et al., 1996), and is responsible for resistance to the Ro1 pathotype of G. rostochiensis (Jacobs et al., 1996; Milczarek et al., 2011; Ramakrishnan et al., 2015).

The Gro1 locus is localized on chromosome VII and contains a family of genes Gro1-1, Gro1-2, Gro1-3, Gro1-4, Gro1-5, Gro1-6, Gro1-8, Gro1-10, Gro1-11, Gro1-12 and Gro1-14, as well as a number of pseudogenes (Barone et al., 1990; Leister et al., 1996; Paal et al., 2004). J. Paal and colleagues showed that the Gro1-4 gene is a monogenic dominant gene responsible for resistance to the Ro1 pathotype of G. rostochiensis and encodes a protein belonging to the TIR-NB-LRR class of proteins. Gro1-4 was introduced into S. tuberosum from the wild potato S. spegazzinii (Ballvora et al., 1995; Gebhardt, Valkonen, 2001; Gebhardt et al., 2004; Paal et al., 2004; Kuhl, 2011; Milczarek et al., 2011; Ramakrishnan et al., 2015).

A number of loci of quantitative traits associated with resistance to cyst nematodes were mapped in the potato genome: Gro1.2, Gro1.3, and Gro1.4 determining resistance to G. rostochiensis were localized on chromosomes X, XI, and III. In this case, S. spegazzinii was the source of resistance (Kreike et al., 1993, 1996).

The Grp1 locus provides a broad spectrum of resistance to both cyst nematodes G. rostochiensis and G. pallida. It has been mapped to chromosome V (Rouppe van der Voort et al., 1998, 2000) and determines resistance to the Ro5 pathotype of G. rostochiensis (Finkers-Tomczak et al., 2009; Milczarek et al., 2011; Ramakrishnan et al., 2015).

A significant number of diagnostic DNA markers have been developed for the H1 gene. Among them are markers CD78 (Pineda et al., 1993), TG689 (Milczarek et al., 2011; Lopez- Pardo et al., 2013), N146, N195 (Mori et al., 2011; Asano et al., 2012), CP113 (Gebhardt et al., 1993; Niewöhner et al., 1995; Skupinová et al., 2002; Milczarek et al., 2011), TG689/ TG689indel12 (Galek et al., 2011), 239E4left (Bakker et al., 2004; Pajerowska-Mukhtar et al., 2009; Milczarek et al., 2011), EM15 (repulsion) and CMI (coupling) (Bakker et al., 2004), 57R (Finkers-Tomczak et al., 2011; Schultz et al., 2012; Milczarek et al., 2014). Markers have also been designed for other genes and QTLs. For example, markers TG69 (Pineda et al., 1993), SCAR-U14, and SCAR-X02 have been developed for the GroV1 gene (Jacobs et al., 1996; Milczarek et al., 2011); markers CP56 and St3.3.2 (Barone et al., 1990; Leister et al., 1996), CP56, CP51(c), GP516(c) were selected for the Gro1 locus (Ballvora et al., 1995; Kuhl, 2011). Markers Gro1-4 (Gebhardt et al., 2004; Paal et al., 2004; Milczarek et al., 2011) and Gro1-4-1 (Asano et al., 2012) were designed for the Gro1-4 gene. For Grp1-QTL, markers GP21 and GP179 (Rouppe van der Voort et al., 1998), TG432 (Finkers-Tomczak et al., 2009; Milczarek et al., 2011) have been developed. The TG63 marker was selected for Gro1.2-QTL (Kreike et al., 1993). Markers Ssp75 and TG30 have been developed for Gro1.3-QTL (Kreike et al., 1993). The Ssp8 marker was designed for Gro1.4-QTL (Kreike et al., 1996).

A number of genes for resistance to wart (S. endobioticum) have been found in potatoes. These are the following genes: Sen1, located on the XI chromosome (Hehl et al., 1999); Sen1- 4 mapped to chromosome IV (Brugmans et al., 2006); locus Sen18-IX, located on chromosome IX; locus Sen2/6/18- I, located on chromosome I (Ballvora et al., 2011); locus Xla-TNL found on chromosome XI (Bartkiewicz et al., 2018); the Sen2 locus mapped to chromosome XI (Plich et al., 2018); the Sen3 locus was mapped on chromosome XI in the same region as the Sen1 gene (Prodhomme et al., 2019); the authors suggested that Sen3 could be either a Sen1 paralogue from the same cluster or an allelic variant of the Sen1 gene.

QTLs responsible for resistance to races 1, 2, 6 and 18 of wart are found on other chromosomes: chromosome I (to race 2), chromosome II (to races 6, 18), chromosome VI (to races 1, 2, 6, 18), chromosome VII (to races 2, 6, 18), chromosome VIII (to races 1, 2, 6, 18), chromosome X (to races 2, 6, 18), chromosome XI (to races 2, 6, 18) (Groth et al., 2013). J.E. Obidiegwu and colleagues also found additional wart resistance loci on chromosomes I, IV, X, XI, and XII that were less influential than the main genes (Obidiegwu et al., 2015). Minor QTLs located on the chromosome X further affect resistance to race 18 of wart (Bartkiewicz et al., 2018).

The Sen1 and Sen1-4 genes determine the resistance to race 1 of the potato wart pathogen; in both cases, resistance is determined by the dominant alleles of the genes. The Sen1 gene is located at the distal part of the long arm of chromosome XI (Hehl et al., 1999; Obidiegwu et al., 2014). However, it should be noted that J.E. Obidiegwu et al. (2015), using genome-wide association studies (GWAS), identified the Sen1/ RSe-XIa multi-allelic locus on potato chromosome XI as the main factor of resistance to four S. endobioticum races (races 1, 2, 6 and 18) (Obidiegwu et al., 2015). The Sen1-4 gene is located on the long arm of chromosome IV at a distance of 5 cM from the centromere (Brugmans et al., 2006).

The Xla-TNL locus on potato chromosome XI is linked to resistance to races 18 and 6 and can be considered as one of the main factors of wart resistance (Bartkiewicz et al., 2018).

The Sen2 locus is mapped to chromosome XI and is a dominant monogenic locus that provides a high level of resistance to eight races of S. endobioticum simultaneously: 1 (D1), 2 (G1), 6 (O1), 8 (F1), 18 (T1), 2 (Ch1), 3 (M1) and 39 (P1). The genetic and physical distances between the Sen1 and Sen2 loci were indirectly estimated at 63 cM and 32 Mbp, respectively (Plich et al., 2018).

Sen3 is a dominant monogenic locus of resistance to races 2, 6, and 18 (Prodhomme et al., 2019). Locus Sen18-IX (chromosome IX) determines resistance to race 18 S. endobioticum, and locus Sen2/6/18-I (chromosome I) to races 2, 6, and 18. A. Ballvora et al. (2011) note that resistances to races 2, 6 and 18 correlate with each other, but are inherited regardless of resistance to race 1.

Several markers have been developed to detect the dominant allele of the Sen1 gene: CP58, GP125 (Hehl et al., 1999), NL25 (Hehl et al., 1999; Bormann et al., 2004; Gebhardt et al., 2006), Sti046, St_At5g16710, GP125 and GP259 (Ballvora et al., 2011). Also, using a genome-wide association studies, a haplotype-specific marker PotVar0067008 associated with Sen1 was identified (Prodhomme et al., 2020).

To identify the Sen18-IX locus, markers GP129, GP101 and STM3023b can be used. The Sen2/6/18-I locus can be diagnosed using markers STM2030, SC176, GP192, GP124, and GP194 (Ballvora et al., 2011). Markers Kc8103 and RK36, located on chromosome XI and linked to the Xla-TNL locus, have shown potential diagnostic value in determining resistance to races 18 and 6 of S. endobioticum (Bartkiewicz et al., 2018). Three markers, 5450_3, 2502_1, and 2502_3, linked to the Sen2 locus were developed (Plich et al., 2018). It is possible to use the markers chr11_1259552 and chr11_1772869 to detect Sen3 (Prodhomme et al., 2019).

The aim of the study was to screen the GenAgro potato collection of the Institute of Cytology and Genetics of the Siberian Branch of the Russian Academy of Sciences (ICG SB RAS) using known diagnostic PCR markers linked to resistance to golden cyst potato nematode and potato wart.

## Materials and methods

Plant material. The research material was the collection of varieties and hybrids of potatoes named the “GenAgro” plant collection of the ICG SB RAS. The collection was represented by 73 varieties and hybrids of potatoes (Solanum tuberosum) (Supplement 1)1. The plants were grown in the field on the territory of the Michurinsky village, Novosibirsk region, from May to August 2017.

Supplementary Materials are available in the online version of the paper: http://vavilov.elpub.ru/jour/manager/f iles/Suppl_Totsky_Engl.pdf


Field tests were carried out according to the following scheme: the number of rows for each genotype was two; the number of plants in a row – 10; row length – 3 m; distance between the rows – 0.75 m; distance between the plants in rows – 0.30 m; planting method – manually (by hand) on furrows, filling furrows with harrows; landing date is the third decade of May.

Agrochemical characteristics of the soil: the content of exchanged potassium 110.00 mg/kg; the amount of exchanged bases 24.19 mg-eq/100 g; hydrolytic acidity 3.23 mg-eq/100 g; exchanged acidity 5.60 mg-eq/100 g; humus content 2.67 %; the content of mobile phosphorus 5.14 mg/kg; the degree of saturation with bases (V) 88.20 %.

Most of the data on resistance to PCN and potato wart were taken from references, namely from the database of the State Register of Selection Achievements Authorized for Use (State Register…, 2019; https://gossortrf.ru/), and from the European Cultivated Potato Database (https://www.europotato.org/). Some of the samples and hybrids for which there were no published data on resistance were evaluated under experimental conditions. Determination of resistance to PCN was carried out in accordance with the methodology recommended by OEPP/EPPO (2006) at the All-Russian Institute of Plant Protection. Potato wart resistance was evaluated according to the Glynn–Lemmerzahl method as described in the EPPO Diagnostic protocol for S. endobioticum (OEPP/EPPO, 2004) at the Russian Potato Research Center.

DNA isolation and PCR analysis. DNA was isolated from the skin of potato tubers using the DNeasy Plant Mini kit (Qiagen, CA, USA) according to the protocol. The concentration and purity of the tested samples were determined using gel electrophoresis and a Nanodrop 2000 apparatus.

Several diagnostic markers most often used in breeding programs were selected for genotyping (Table 1). These markers were associated with R-genes that determine resistance to race 1 of potato wart (S. endobioticum) and Ro1 pathotype of potato cyst nematode (G. rostochiensis).

**Table 1.**
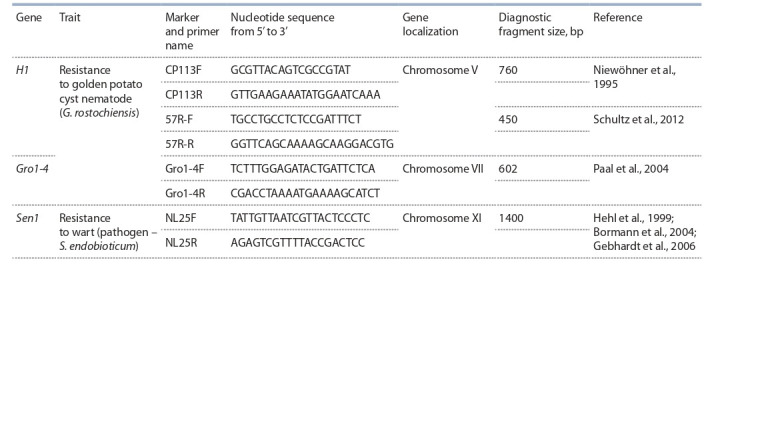
DNA markers used for collection screening

Two markers, 57R and CP113, associated with the H1 resistance gene, and the Gro1-4 marker, associated with the Gro1-4 resistance gene, were selected to identify PCN resistance genes (see Table 1). The SCAR PCR marker CP113-5’2/ CP113-3’2 was proposed by J. Niewöhner et al. (1995) based on the RFLP marker CP113. Amplification of DNA of resistant genotypes using this marker formed product with a 760 bp length. The 57R marker was proposed by L. Schultz et al. (2012). Amplification of DNA of resistant genotypes formed product with a 450 bp length. SCAR PCR marker Gro1-4 was developed by J. Paal et al. (2004) based on the RFLP marker Gro1. Amplification of DNA of resistant genotypes formed product with a 602 bp length.

The NL25 marker was proposed by R. Hehl et al. (1999) when mapping the Sen1 gene. C.A. Bormann et al. (2004) and C. Gebhardt et al. (2006) used this marker for markerassisted selection (see Table 1). Amplification produces one or two fragments of 1200 or 1400 bp lenght. The presence of the dominant Sen1 allele is determined by the presence of a 1400 bp fragment.

PCR was carried out in a 20 μL reaction mixture containing 100 ng of DNA, 67 mM Tris-HCl (pH 8.8), 1.8 mM MgCl2, 0.01 % Tween 20, 0.2 mM each dNTP, 0.25 μM forward and reverse specific primers, 1 unit Taq DNA polymerase.

Two types of amplification programs (SSR55 and SSR60) represented the time-temperature profile of PCR. SSR55: (1) first cycle: 94 °C – 2 min; (2) the next 45 cycles: 94 °С – 1 minute, 55 °С – 1 minute and 72 °С – 2 minutes; (3) one cycle of 5 minutes at 72 °C (Gro1-4). SSR60: (1) first cycle: 94 °C – 2 min; (2) the next 45 cycles: 94 °С – 1 minute, 60 °С – 1 minute and 72 °С – 2 minutes; (3) one cycle of 5 minutes at 72 °C (NL25, CP113, 57R).

The analysis of the obtained PCR products was carried out by electrophoresis in a 2 % agarose gel. The results were documented using a Molecular Imager Gel Doc XR System (BioRad) using UV light.

Statistical processing of the data was carried out using Spearman’s correlation coefficient; for calculations, the STATISTICA program was used. The diagnostic efficiency, sensitivity, specificity and predictive value were calculated using the MedCalc software

https://www.medcalc.org/

Diagnostic efficiency was defined as the proportion of correct test results in the total number of test results, or the sum of true positive and true negative test results divided by the total number of test results. The sensitivity was calculated as the number of resistant samples identified using a DNA marker divided by the total number of resistant samples. Specificity is the number of susceptible samples identified by the DNA marker divided by the total number of susceptible samples. Positive predictive value was defined as the proportion of correct positive diagnostic test results.

## Results

Among 73 samples selected for genotyping, 35 were resistant to PCN, 37 samples were susceptible, and in one sample, resistance to nematodes was unknown (Table 2). 69 samples were resistant to wart, 3 samples were susceptible to disease, the resistance of one sample was unknown (see Table 2).

**Table 2.**
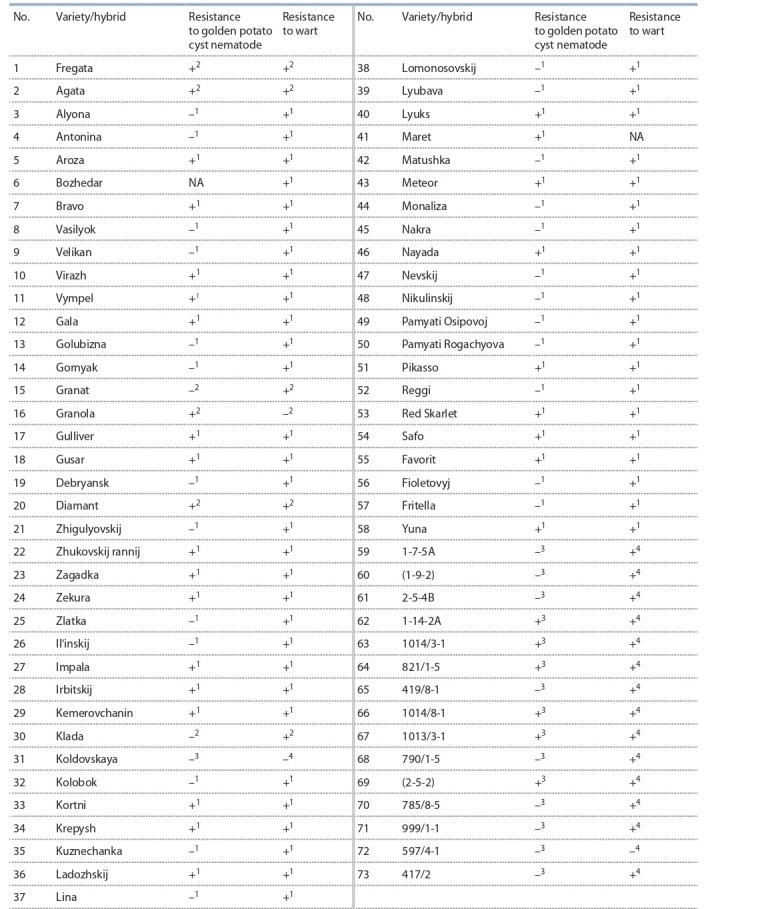
Resistance of varieties and hybrids of potatoes to nematodes and wart Notе. 1 State Register of Selection Achievements Authorized for Use; 2 The European Cultivated Potato Database; 3 All-Russian Institute of Plant Protection; 4 Russian Potato Research Center; NA – no data.

**Genotyping of varieties and hybrids using markers designed for resistance to PCN** The 57R marker is found in 85.7 % of resistant samples, as well as in 13.5 % of susceptible ones (Table 3; Supplement 2, Fig. 1–6; Supplement 3). Some mismatches can be observed due to the absence of linkage of the 57R marker with the H1 resistance gene in a number of samples. The second reason for the mismatches can be explained by the presence of other resistance genes in samples that do not carry the 57R marker. The diagnostic efficiency of the 57R marker, which is expressed as the percentage of true (both positive and negative) test results to the total number of results obtained, was 86.11 %. The diagnostic sensitivity of the used marker, which shows the number of resistant samples identified using the DNA marker divided by the total number of resistant samples, was 85.71 %. The diagnostic specificity, which is the number of susceptible samples identified by the DNA marker divided by the total number of susceptible samples, was 86.48 %. The predictive value of a positive result, showing the proportion of correct positive diagnostic test results, was 85.71 %. Calculation of the Spearman correlation coefficient (Spearman R = 0.722008, p = 0.000000, p < 0.05) showed a significant correlation between resistance and the 57R marker.

**Table 3.**
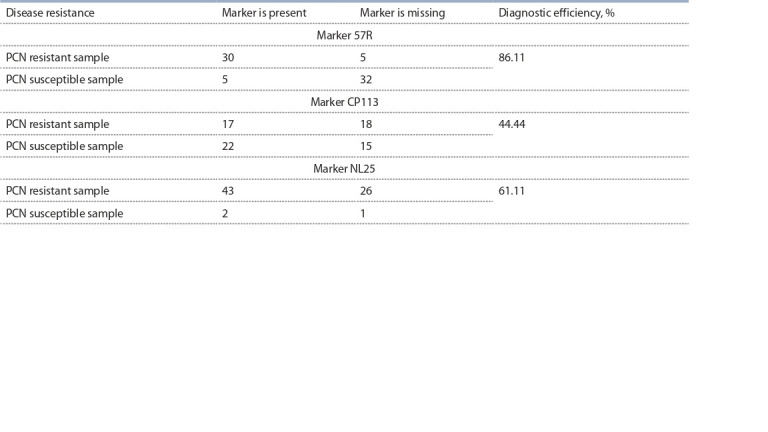
Results of screening of potato cultivars and hybrids collection for PCN resistance using the 57R marker and for wart resistance using the NL25 marker

The CP113 marker is found in only 48.6 % of resistant accessions, while the marker is present in 62.9 % of susceptible genotypes (see Table 3; Supplement 2, Fig. 7; Supplement 3). These results can be regarded as the absence of linkage of the marker with the H1 resistance gene in many samples of the potato collection. The diagnostic efficiency of the CP113 marker was only 44.44 %. Diagnostic sensitivity was 48.57 %. Diagnostic specificity accounted for 40.54 %. The predictive value of a positive result, indicating the probability of resistance presence if the test shows a positive result when CP113 marker was used, was equal to 43.58 %. Spearman’s correlation coefficient (Spearman R = –0.109218, p = 0.361104, p < 0.05) in this case showed no significant correlation between resistance and DNA marker. The use of such a marker when screening a population to search for resistant samples is not advisable.

29 samples were analyzed using the Gro1-4 marker. The diagnostic fragment was amplified in only 5 samples. Correspondence of the presence of the marker in the resistant sample was observed only in 1 case out of 5. In other cases, the marker was found in the samples susceptible to the disease.

The data obtained show that when screening populations for resistance to PCN, it is advisable to use the 57R marker.

**Genotyping of varieties and hybrids using markers linked to resistance to potato wart** The NL25 marker is found in 62.3 % of resistant samples, however, the marker is present in two of the three susceptible genotypes (see Table 3, Supplement 4). This can be explained by the processes of crossing over and by the fact that in a number of samples the linkage of the marker and the resistance gene is not observed; however, the small number of sensitive samples does not allow sufficiently assessing the applicability of the marker for breeding. The marker is absent in 27 samples and only in one case we observe the absence of a marker in the susceptible sample, in the other cases the marker is absent in the resistant samples. This can be explained by the presence of another resistance gene that is not linked to the NL25 marker.

The diagnostic efficiency of resistance using the NL25 marker was 61.11 %. The diagnostic sensitivity turned out to be at 62.31 %. The diagnostic specificity was only 33.33 %. However, the predictive value of a positive result, showing the proportion of correct positive diagnostic test results, when using the NL25 marker was equal to 95.55 %. It should be noted that such results are associated with the fact that the set of samples contained only three sensitive samples, and two of them showed the presence of the NL25 marker. Spearman’s correlation coefficient (Spearman R = –0.017946, p = 0.881061, p < 0.05) in such situation showed the absence of significant correlations.

Despite the fact that the NL25 marker is often used in screening and marker selection, a study in our set of samples showed that its use does not guarantee a reliable result.

## Discussion

In our study, 13 resistant to golden potato nematode samples that had both markers (57R and CP113) linked to the H1 nematode resistance gene were found. In addition, there are 8 genotypes resistant to nematodes and wart and carrying both the 57R and CP113 markers linked to the H1 nematode resistance gene and the NL25 marker linked to the Sen1 wart resistance gene. There is also one sample (Safo) in the population that is resistant to wart and nematodes and carries all three markers 57R, CP113, Gro1-4, linked to nematode resistance, and marker NL25, linked to wart resistance.

## DNA markers of wart resistance

The NL25 marker linked to the Sen1 gene, which provides resistance to pathotype 1 of potato wart, is successfully used in the practice of marker-oriented selection. So, C. Gebhardt and colleagues reported that after screening 17 plants in two families of segregating populations using the NL25 marker, 14 genotypes with the marker were identified. All these plants were found to be resistant to pathotype 1 S. endobioticum. Some were also resistant to pathotype 2 and/or pathotype 6 (Gebhardt et al., 2006).

The effectiveness of this marker is also reported by O.Y. Antonova and colleagues who analyzed 98 varieties using the NL25 marker. A diagnostic component was found in 95 studied wart-resistant varieties, while it was not found in three susceptible varieties. This shows a high level of correlation between the presence or absence of the marker and the resistance and sensitivity of the genotype to wart, respectively (Antonova et al., 2016).

However, A. Khiutti and colleagues, when screening 52 genotypes using the NL25 marker, found that 39 samples (both sensitive and resistant genotypes) had the same nondiagnostic fragment, 12 genotypes did not have amplification of the NL25 marker fragments. Only 5 out of 52 genotypes had a diagnostic fragment indicating the presence of a resistance gene. Four of these five accessions were resistant, but one genotype was found to be sensitive; most resistant genotypes did not have a 1400 bp diagnostic fragment predicting a resistant phenotype (Khiutti et al., 2012).

Our analysis also did not allow us to speak about the reliability of using the NL25 marker for screening resistant varieties.

## DNA markers of resistance to PCN

Using the Gro1-4 marker in a segregating population, C. Gebhardt and colleagues found that all 45 plants carrying this marker linked to the Gro1 gene were resistant to the Ro1 pathotype of G. rostochiensis (Gebhardt et al., 2006).

C. Gebhardt and colleagues in 1993 found in a segregating population that the CP113 marker is linked to the H1 gene so strongly that it has zero recombination (Gebhardt et al., 1993). However, D. Milczarek and colleagues (2011) reported that the CP113 marker was amplified for all tested varieties, resistant and sensitive, and was unsuitable for the selection of resistant clones. A similar picture is observed in our work.

The 57R SCAR marker was tested in a mapping population, where it was linked to the H1 locus and nematode resistance (Finkers-Tomczak et al., 2011). Later L. Schultz and colleagues reported that they analyzed two independent populations of 281 and 122 potato samples with known resistance/sensitivity using the 57R SCAR marker. When screening the first population, the 57R marker revealed a correspondence between genotype and phenotype, 89 out of 90 resistant varieties had an allele associated with resistance. Only one resistant variety, in which no marker amplification was observed, became an exception. None of the 191 PCN susceptible varieties had an allele predicting resistance. Then another independent population of 122 varieties was screened. All varieties showed complete correspondence between resistance to G. rostochiensis and the presence/absence of the 57R allele, corresponding to the presence of the resistance gene (Schultz et al., 2012).

O.Y. Antonova et al. (2016) identified the 57R marker in 33 (30.3 %) of 109 breeding varieties they studied. The overwhelming majority of the varieties with the diagnosed 57R fragment were resistant or weakly affected by the nematode. The correspondence between resistance and the presence of a diagnostic fragment was high – 93.5 %. At the same time, only four genotypes with the Gro1-4 marker were identified: two resistant varieties, one weakly affected variety and one susceptible. All these four varieties, along with the Gro1-4 marker, also possessed the H1 gene markers – 57R, TG689, N146, N195 (Antonova et al., 2016).

In the work of N.S. Klimenko et al. (2017) showed the presence of the 57R marker in 24 out of 103 samples, while the marker was found in 15 resistant and 2 susceptible samples. It was shown that the correlation between the presence of at least one marker of the H1 gene and the data on the nematode resistance of varieties was +0.92 (Klimenko et al., 2017).

T.A. Gavrilenko et al. (2018) showed that out of 39 samples of the studied set of samples, 15 had a dominant allele of the H1 gene (based on a number of DNA markers), and two varieties had dominant alleles of both H1 and Gro1-4 genes. At the same time, none of the markers was identified in the remaining 22 genotypes. Comparison of these results with resistance to G. rostochiensis (pathotype Ro1) showed that all accessions with H1 gene markers are nematode resistant, while varieties affected by G. rostochiensis did not have these markers (Gavrilenko et al., 2018). This high correlation shows the reliability of the markers used in the study, which can be used to select resistant samples.

It should be noted that the saturation of the genotype with genes of resistance to the nematode does not affect its economically valuable traits. At the same time, there is a strong link between the presence of the marker and resistance. So, in the study of D. Milczarek and colleagues in 2014, the relationship between the presence of markers TG689 and 57R linked to the H1 gene, which determines resistance to the nematode G. rostochiensis, and valuable agricultural traits is presented. Clones with these markers had a higher total yield of tubers and total starch yield than clones without markers. There was no negative association between marker presence and quality. All 347 seedlings obtained after three crosses were genotyped using both markers and phenotypically evaluated for resistance to the Ro1 pathotype of G. rostochiensis. Of these, 316 (i. e. 91 %) and 325 (94 %) clones were resistant and carried the TG689 or 57R markers (Milczarek et al., 2014).

## Conclusion

In general, our data on the 57R marker are quite close to the results described above and confirm the high reliability of the work of this marker, which suggests the need to use this marker when selecting samples resistant to PCN.

## Conflict of interest

The authors declare no conflict of interest.
